# Evaluation of antibody responses to the early transcribed membrane protein family in *Plasmodium vivax*

**DOI:** 10.1186/s13071-019-3846-4

**Published:** 2019-12-19

**Authors:** Seong-Kyun Lee, Jin-Hee Han, Ji-Hoon Park, Kwon-Soo Ha, Won Sun Park, Seok-Ho Hong, Sunghun Na, Yang Cheng, Eun-Taek Han

**Affiliations:** 1 Department of Medical Environmental Biology and Tropical Medicine, School of Medicine, Kangwon National University, Chuncheon, Gangwon-do 24341 Republic of Korea; 2 Department of Cellular and Molecular Biology, School of Medicine, Kangwon National University, Chuncheon, Gangwon-do 24341 Republic of Korea; 3 Department of Physiology, School of Medicine, Kangwon National University, Chuncheon, Gangwon-do 24341 Republic of Korea; 4 Department of Internal Medicine, School of Medicine, Kangwon National University, Chuncheon, Gangwon-do 24341 Republic of Korea; 5 Department of Obstetrics and Gynecology, Kangwon National University Hospital, Chuncheon, Gangwon-do 24341 Republic of Korea; 6 Department of Public Health and Preventive Medicine, Laboratory of Pathogen Infection and Immunity, Wuxi School of Medicine, Jiangnan University, Wuxi, Jiangsu People’s Republic of China

**Keywords:** Malaria, *Plasmodium vivax*, ETRAMP, IgG antibody response, *P. vivax* patients

## Abstract

**Background:**

Malaria parasites form intracellular membranes that separate the parasite from the internal space of erythrocytes, and membrane proteins from the parasites are exported to the host *via* the membrane. In our previous study, *Plasmodium vivax* early transcribed membrane protein (PvETRAMP) 11.2, an intracellular membrane protein that is highly expressed in blood-stage parasites, was characterized as a highly immunogenic protein in *P. vivax* malaria patients. However, the other PvETRAMP family proteins have not yet been investigated. In this study, PvETRAMPs were expressed and evaluated to determine their immunological profiles.

**Methods:**

The protein structure and amino acid alignment were carried out using bioinformatics analysis software. A total of six PvETRAMP family proteins were successfully expressed and purified using a wheat germ cell free protein expression system and the purified proteins were used for protein microarray and immunization of mice. The localization of the protein was determined with serum against PvETRAMP4. IgG subclasses were assessed from the immunized mice.

**Results:**

*In silico* analysis showed that *P. vivax* exhibits nine genes encoding the ETRAMP family. The ETRAMP family proteins are relatively small molecules with conserved structural features. A total of 6 recombinant ETRAMP proteins were successfully expressed and purified. The serum positivity of *P. vivax* malaria patients and healthy individuals was evaluated using a protein microarray method. Among the PvETRAMPs, ETRAMP4 showed the highest positivity rate of 62%, comparable to that of PvETRAMP11.2, which served as the positive control, and a typical export pattern of PvETRAMP4 was observed in the *P. vivax* parasite. The assessment of IgG subclasses in mice immunized with PvETRAMP4 showed high levels of IgG1 and IgG2b. PvETRAMP family proteins were identified and characterized as serological markers.

**Conclusions:**

The relatively high antibody responses to PvETRAMP4 as well as the specific IgG subclasses observed in immunized mice suggest that the ETRAMP family is immunogenic in pathogens and can be used as a protein marker and for vaccine development.
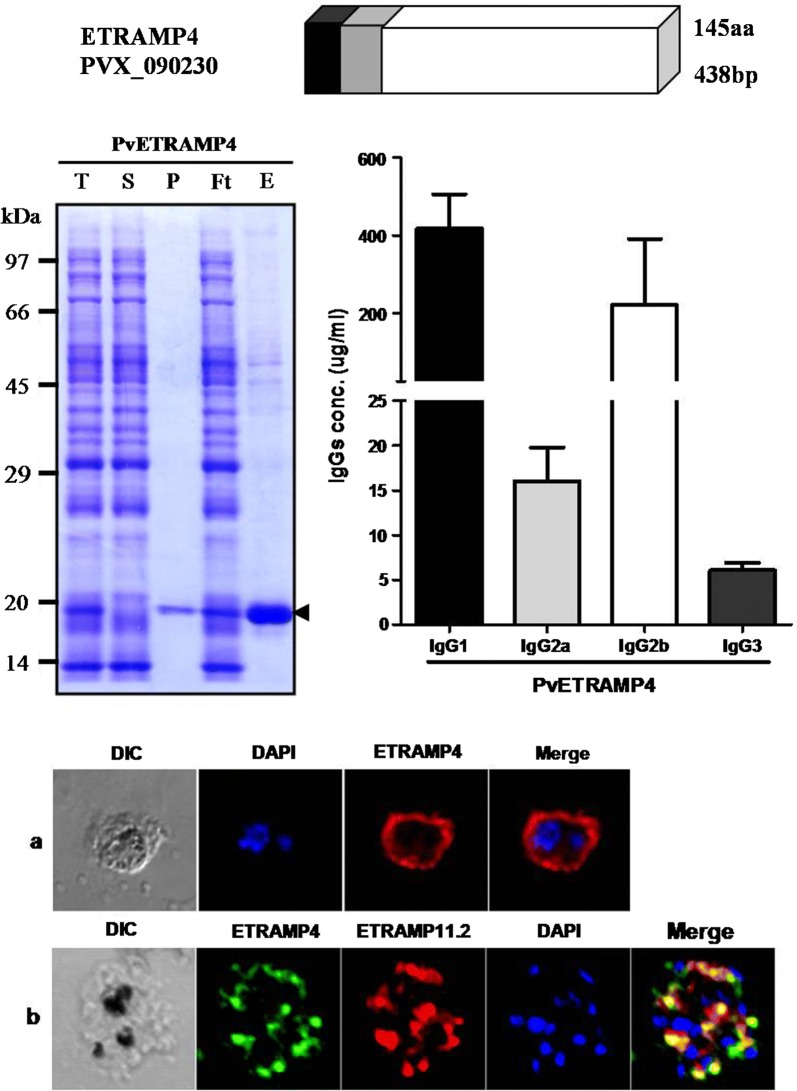

## Background

Malaria is a major global public health problem and causes pathogen-specific mortality. Approximately three billion people from approximately 100 countries are exposed to the five human-infectious malaria parasites, resulting in 219 million malaria infections in 2017 [1, 2]. In order to eliminate the parasite from the host, malaria-specific antibodies play essential roles in acquired immunity *via* the phagocytosis and opsonization of parasites [3], and the importance of immunity is evident in experimental animal models as well as passive transfer studies in which antibodies from a parasite-infected donor patient have been used to effectively treat patients with severe malaria [4, 5]. Moreover, the identification and the evaluation of antibodies raised against unknown merozoite antigens involved in parasite survival is also necessary for the development of serological markers as well as a vaccine [6–8]. For this reason, the development of omics techniques and high-throughput screening systems involving cell-free protein synthesis technology has led to the identification of numerous vaccine candidates and has extended the possibilities for investigating serological markers that induce an immune response in endemic areas of *Plasmodium falciparum* and *P. vivax* [9–12].

The early transcribed membrane protein (ETRAMP) family was identified first in human-infectious malaria parasites, followed by rodent malaria parasites, and shows conserved structural features such as the presence of a signal peptide at the N terminus and a charged domain in the C-terminal region [13–17]. Interestingly, most ETRAMPs are expressed in stage-specific expression patterns during the parasite life cycle, and they mostly localize to the parasitophorous vacuole membrane (PVM), which spatially separates the parasite from the cytosol of erythrocytes in infected RBCs and mediates the free passage of molecules, probably through membranous pores such as the *Plasmodium* translocon for exported proteins (PTEX) and exported protein 1 (EXP1) [18–22]. On the other hand, the *P. berghei* ETRAMP family member small exported protein 2 (SEP2), localizes to membranous compartments of the ookinete and is released during gliding motility in the sporozoite, indicating that the protein family is involved in not only blood stage but also sexual stage [23].

In our previous studies, 232 blood stage-specific proteins of *P. vivax* were screened using *P. vivax* patient serum samples to evaluate the immune response and explore vaccine candidates using a protein array method, and PvETRAMP11.2 showed a relatively high antibody responses under relapse and reinfection of *P. vivax* according to serological analysis, indicating that the PvETRAMP family is likely to be immunogenic in *P. vivax* patients [24–26]. Therefore, in the present study, the other ETRAMPs of *P. vivax* were expressed for evaluation of serum reactivity to *P. vivax*-infected patient sera and profiling of the IgG subclass phenotype.

## Methods

### Bioinformatics analysis

The genes encoding *pvetramps* were retrieved from PlasmoDB (http://plasmodb.org), along with their predicted transcriptional expression levels in blood-stage parasites. Alignments of the corresponding amino acid sequences were carried out with Clustal W2 [27]. The Simple Modular Architecture Research Tool (SMART) (http://smart.embl-heidelberg.de/) and the TMHMM Server v. 2.0 (http://www.cbs.dtu.dk/service/TMHMM) were used for the prediction of signal peptides and transmembrane domains [28–31].

### Sample and serum preparation

Blood samples that were confirmed to be either *P. vivax* positive or negative *via* testing with a rapid diagnosis test kit and microscopy were collected in the Republic of Korea with approval from the Institutional Review Board at Kangwon National University (IRB No. 2017–05–009–001). The collected whole blood samples were centrifuged to separate the serum and packed cells.

### Cloning and protein expression of PvETRAMPs

The six *pvetramps* were amplified by PCR from genomic DNA isolated from Korean *P. vivax* malaria patients. The primers used for PCR are described in Table 1. The six amplified genes were cloned into the *Xho*I and *BamH*I sites of the pEU-E01-His-Tev-N2 plasmid vector (CellFree Sciences, Matsuyama, Japan) for protein expression using in-fusion cloning (Clontech, Palo Alto, CA, USA). The nucleotide sequence of *pvetramps* in the pEU plasmid DNA vector was confirmed by sequence analysis (Genothech, Daejeon, Korea). The pEU plasmid DNAs were purified with a Midiplus ultrapure plasmid extraction system (Viogene, Taipei, Taiwan) and were used for protein expression as described in a previous report [32]. The recombinant proteins were purified using a Ni-nitrilotriacetic acid (Ni-NTA) affinity chromatography column (Qiagen, Hilden, Germany) [33]. The purified proteins were confirmed by 12% SDS-PAGE and western blot analysis under reducing conditions.

**Table 1:** Primer sequence information for *pvetramps* for cloning into the pEU expression vector

Gene ID	ORF (bp)	Expression region sequence (bp)	Genic-specific sense primer (5′–3′)	Genic-specific anti-sense primer (5′–3′)
PVX_090230	438	238–438 (201)	**GGGCGGATATCTCGAG**TACTACAAGCAGAAGAAGAGCAAGG	**GCGGTACCCGGGATCC**TCATTTGGATG GGGGGGT
PVX_096070	549	79–627 (549)	**GGGCGGATATCTCGAG**CATGTAAATAACAAGCCCCACG	**GCGGTACCCGGGATCC**TTAAGATTCTTGTGGGGTTTCTG
PVX_086915	564	76–564 (489)	**GGGCGGATATCTCGAG**AATTTATTTCTGGAAAAAGTGAAAGC	**GCGGTACCCGGGATCCTTA**TTTACTGTTCGGAATTAAGCTTG
PVX_088870	519	76–519 (444)	**GGGCGGATATCTCGAG**GAGAATGTGGTAAAGAAGAAAGTCCT	**GCGGTACCCGGGATCC**TCACGTTTGAGTGTCACCAG
PVX_001715	504	64–501 (438)	**GTTCCGCGTGGATCCATG**GAGGAAGTGAAGGCCGTCT	**ATGCGGCCGCTCGAG**TATAATGGGAGATCCCATAACA
PVX_003565	333	67–333 (267)	**GGGCGGATATCTCGAG**TTCTACAATAATGTTGTAGCAGGAAAG	**GCGGTACCCGGGATCC**TTATTGGATGTTGCTGCCTTT

*Note*: The vector sequences are shown in bold

### Evaluation of antibody responses against six recombinant PvETRAMPs in *P. vivax* malaria patients or healthy individuals using a protein microarray

The preparation of amine-coated slides was described in a previous report [25]. Serum samples from *P. vivax* malaria patients or healthy individuals were used to evaluate the antibody reactivity of PvETRAMPs. A recombinant protein (100 ng in each protein) was spotted in each well of a slide, followed by incubation for 2 h at 37 °C. After washing the slide with phosphate-buffered saline-Tween (PBST, 0.1%), the slide was blocked with 5% bovine serum albumin (BSA) in PBS for 1 h at 37 °C. The slide was probed with serum (1:10 dilution) and incubated for 1 h at 37 °C. Alexa Fluor 546-conjugated goat anti-human IgG (10 ng/μl; Invitrogen, Carlsbad, CA, USA) was spotted on the slide, followed by incubation for 1 h at 37 °C for visualization. The slide was scanned in a fluorescence scanner (Perkin Elmer, Boston, MA, USA) [34]. The mean fluorescence intensity (MFI) of each spot was quantified using ScanArray Express software version 4.0 (Perkin Elmer). The cut-off MFI value was determined as the MFI of serum from a healthy individual plus two standard deviations [33].

### Indirect immunofluorescence assay (IFA)

Slides smeared with an enriched schizont-stage of *P. vivax* from patient blood samples were fixed with ice-cold acetone for 3 min, dried, and stored at − 80 °C until use. The slides were blocked with 5% BSA in PBS at 37 °C for 30 min and washed with PBS. The slides were incubated with a 1:50 diluted primary antibody (mouse anti-PvETRAMP4 and rabbit anti-PvETRAMP11.2) at 37 °C for 1 h. The slides were stained with Alexa Fluor 488-conjugated anti-mouse IgG, Alexa Fluor 568-conjugated anti-mouse IgG and Alexa Fluor 568-conjugated goat anti-rabbit IgG secondary antibodies (Invitrogen) and 4’,6-diamidino-2-phenylindole (DAPI; Invitrogen) at 37 °C for 30 min. The stained slides were mounted in ProLong Gold antifade reagent (Invitrogen) and visualized under a confocal laser scanning FV200 microscope (Olympus, Tokyo, Japan). The captured images were analyzed using FV10-ASW 3.0 viewer software.

### Immunization and IgG subclass profiling

To induce IgGs against PvETRAMP4, 20 μg of recombinant protein was injected into 5-week-old female BALB/c mice (Daehan Biolink Co., Eumsung, Korea) with Freund’s complete adjuvant, followed by an incomplete adjuvant. The injections were performed a total of three times at 2-week intervals. Enzyme-linked immunosorbent assays (ELISAs) were used for the profiling of IgG subclasses produced against PvETRAMP4 in mice [35]. Ninety-six-well ELISA plates (Costar, Corning, NY) were coated with purified mouse IgG1, IgG2a, IgG2b, and IgG3 (Invitrogen) and incubated with immunized mouse sera diluted 1:1000 in PBST. Horse-radish peroxide-conjugated anti-mouse IgG1, IgG2a, IgG2b, and IgG3 antibodies (Invitrogen) at 1:1000, 1:1000, 1:2000, and 1:1000 dilutions, respectively, were used to detect each isotype antibody. The reaction was developed by adding 100 μl of diluted 3, 3′, 5, 5′-tetramethylbenzidine single solution (Invitrogen, Rockford, IL) for 15 min at 37 °C, then stopped with 100 μl of 1N HCl solution. The intensity was measured and calculated from the log–log curve fit.

### Statistical analysis

The data were analyzed with GraphPad Prism (GraphPad Software, San Diego, CA) and Microsoft Excel 2016 (Microsoft, Redmond, WA). Unpaired t*-*tests were used to compare the differences between the means of each group. Values of *P *<0.05 were considered statistically significant. Sensitivity was calculated based on the percentage of patients exhibiting values above the cut-off MFI, and specificity was calculated based on the percentage of healthy patients presenting values below the cut-off MFI.

## Results

### Identification of PvETRAMP proteins

**Fig. 1:**
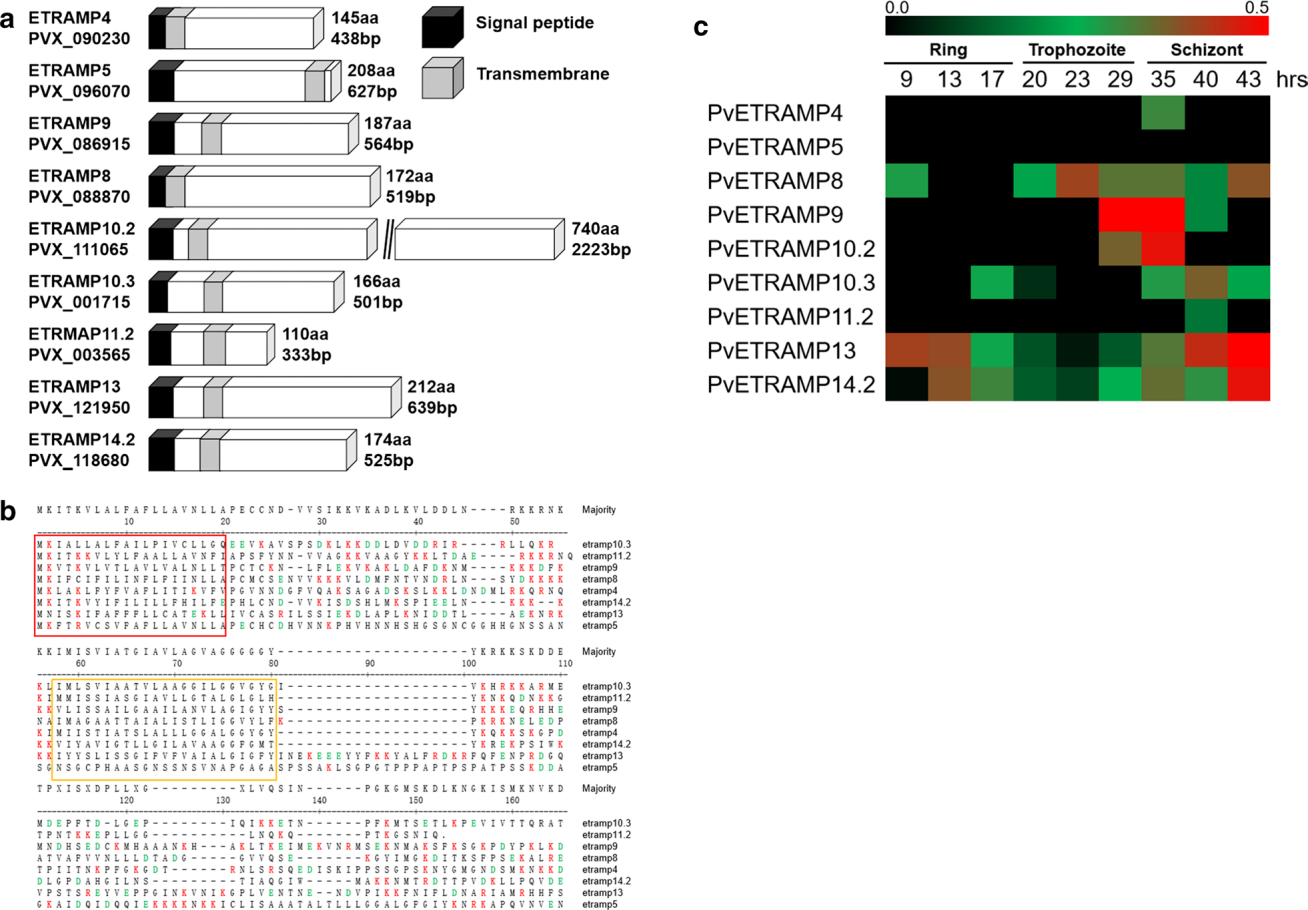
Schematic structures and amino acid alignments of the PvETRAMP family. **a** Schematic structure of the PvETRAMP family. A total of nine PvETRAMPs were identified. The signal peptide and transmembrane domain are indicated by a black box and a gray box, respectively. **b** Analysis of conserved sequence regions in PvETRAMPs. Eight PvETRAMP proteins showed the conserved domains, including the signal peptide (red box) and transmembrane domain (yellow box). **c** Predicted transcription levels of PvETRAMPs

Nine proteins in the ETRAMP family were found in *P. vivax*, and the proteins were named according to their orthologues in *P. falciparum.* The PvETRAMPs were relatively small molecules except for PvETRAMP10.2, showing sizes of 11.9–25.0 kDa and 110–212 amino acids (Table 2, Fig. 1a). However, all PvETRAMPs presented high conservation of a signal peptide at the N terminus and one or two transmembrane domain(s) flanked by highly charged domain(s) containing amino acids such as lysine (K) and aspartic acid (D), leading most PvETRMPs to exhibit high pI values (Fig. 1a, b). In the transcriptome analysis, PvETRAMP4, 9, and 10.2 were predicted to be highly transcribed at the late trophozoite and early schizont stages, and PvETRAMP13 and 14.2 showed high transcription levels at the late schizont stage, indicating that expression of PvETRAMPs is mostly stage specific during intraerythrocytic development (Fig. 1c).

**Table 2:** The genetic information for the *etramp* family in *Plasmodium vivax*

Gene	PlasmoDB gene ID				SP	TM	kDa	ORF (bp)	pI
	*P. vivax* (Sal-1)	*P. vivax* (P01)	*P. falciparum* (3D7)	*P. knowlesi* (strain H)					
*etramp4*	PVX_090230	PVP01_0532300	PF3D7_0423700	–	Y	TM*2	15.8	438	10.8
*etramp5*	PVX_096070	PVP01_0004210	PF3D7_0532100	PKNH_031400	Y	TM	21.0	627	7.3
*etramp9*	PVX_086915	PVP01_0734800	PF3D7_0936100	PKNH_0734700	Y	TM*2	21.5	564	10.3
*etramp8*	PVX_088870	PVP01_0504800	PF3D7_0829600	PKNH_1323800	Y	TM*2	18.9	519	9.6
*etramp10.2*	PVX_111065	PVP01_0618300	PF3D7_1033200	PKNH_0618000	Y	TM	78.9	2226	4.1
*etramp10.3*	PVX_001715	PVP01_0602100	PF3D7_1016900	PKNH_0601100	Y	TM	18.2	504	9.1
*etramp2/etramp11.2*	PVX_003565	PVP01_0422600	PF3D7_0202500 PF3D7_1102800	PKNH_0418600	Y	TM*2	11.9	333	11.0
*etramp13*	PVX_121950	PVP01_1403100	PF3D7_1302200	PKNH_1402400	Y	TM*2	25.0	642	8.9
*etramp14.2*	PVX_118680	PVP01_1271000	PF3D7_1476100	PKNH_1246400	Y	TM*2	20.0	528	9.9

*Abbreviations*: SP, signal peptide; TM, transmembrane; pI, isoelectric point; ORF, open reading frame

### Expression and purification of recombinant PvETRAMP proteins from a wheat germ cell-free system

**Fig. 2:**
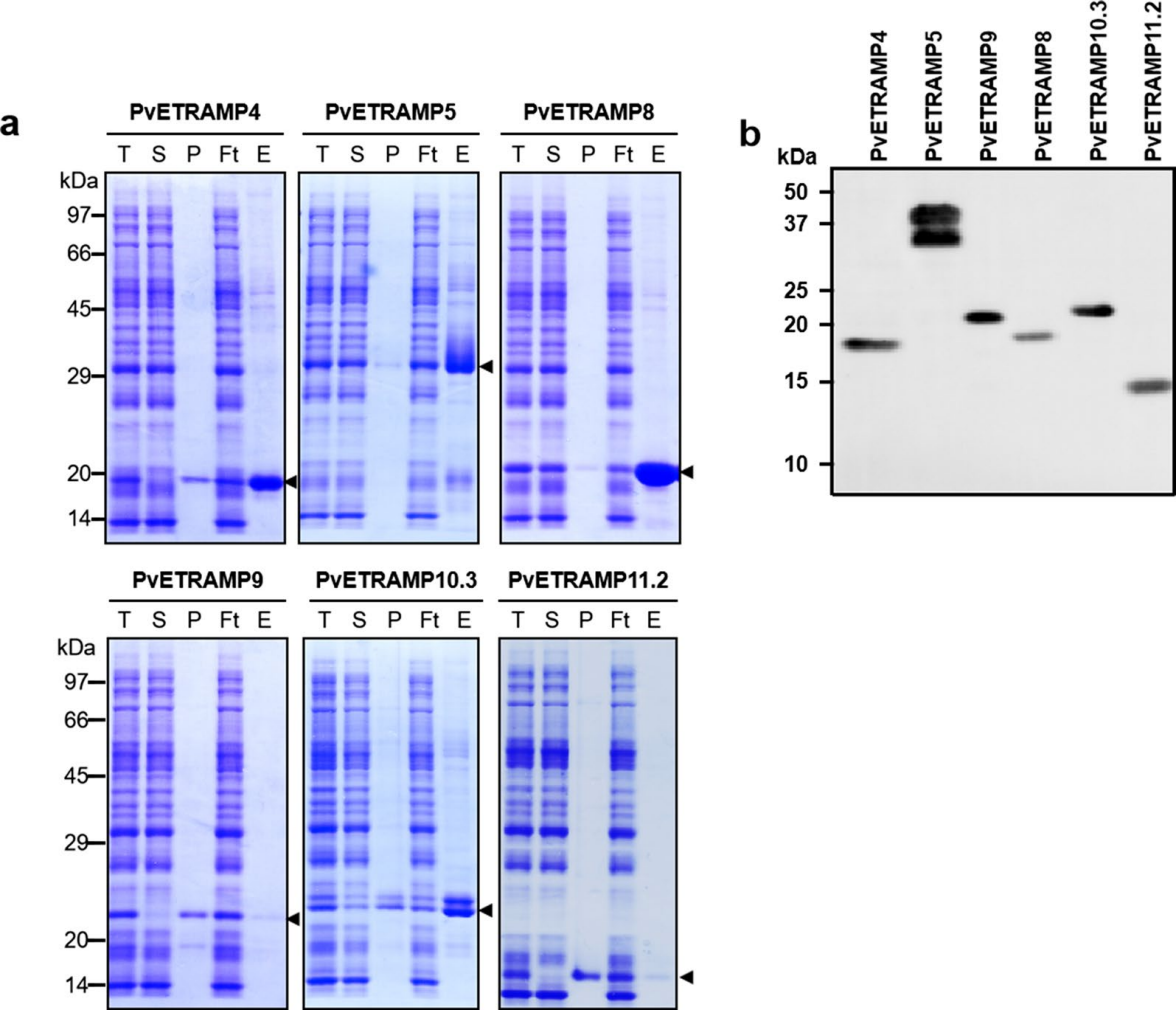
Recombinant protein expression and purification of PvETRAMPs in a wheat germ cell-free expression system. **a** SDS-PAGE of purified PvETRAMPs. Arrows indicate purified PvETRAMP proteins. **b** Western blot of PvETRAMPs with an anti-penta-His antibody. *Abbreviations*: M, protein marker; T, total fraction; S, soluble fraction; P, pellet fraction; Ft, flow-through; E, elution fraction

A total of six PvETRAMP proteins were successfully expressed and purified using a wheat germ cell-free system, and their purity was confirmed by SDS-PAGE and western blot analyses (Fig. 2). The specific bands observed in the elution fraction indicated that the recombinant proteins were purified in a soluble form. However, the expression or solubility rate of PvETRAMP9 and 11.2 was considerably lower than those of the others. Two proteins, PvETRAMP5 and PvETRAMP10.3, had different banding patterns in the SDS-PAGE and immunoblot results, which was thought to be non-specific proteins co-expressed with target proteins. Most of the recombinant proteins were observed at slightly greater or lower sizes than their expected molecular weights, which might be due to the pI values of proteins, and these results were consistent with PfETRAMPs (Table 2) [13].

### Humoral immune response evaluation of PvETRAMPs

**Fig. 3:**
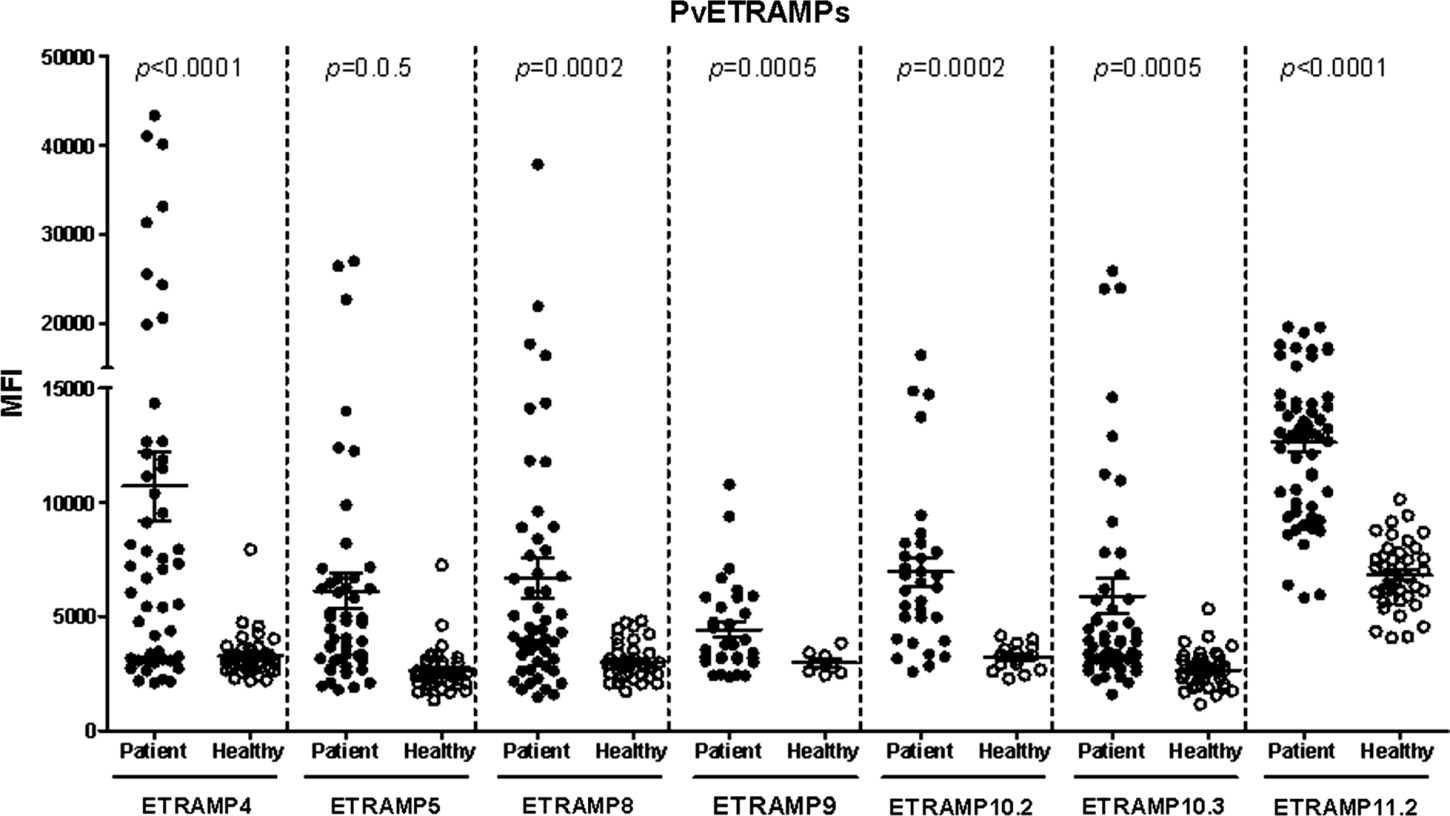
IgG antibody responses to PvETRAMPs in the sera of *Plasmodium vivax* patients and malaria-naïve individuals. PvETRAMPs were reacted with the sera of *P. vivax* malaria patients or healthy individuals from the ROK. *P*-values were calculated with unpaired t-tests. The bar indicates the mean plus SD

A protein array method was exploited to evaluate the humoral immune responses against PvETRAMPs (Fig. 3). All PvETRAMPs showed high specificity (> 95%), indicating that they can be used for differentiating infected and non-infected humans. There were significant antibody reactivity differences between patients and healthy individuals (*P* < 0.05) for most PvETRAMPs, except for PvETRAMP9 (*P *= 0.05) (Table 3). The sera from *P. vivax-*infected individuals to PvETRAMPs showed significantly higher mean fluorescence intensity (MFI) values than those from malaria-naïve individuals. The highest seropositivity was found for PvETRAMP11.2 (76.8%), followed by PvETRAMP4 (62%), which was used for further study.

**Table 3:** Prevalence, 95% confidence intervals, and mean fluorescence intensities of IgG responses to PvETRAMPs in *P. vivax* malaria patients and healthy individuals

Antigen (*P. vivax*)	Sample (*n*)	No. of samples		Sensitivity (%)/Specificity (%)	95% CI (%)	MFI	Cut-off value^a^	*t-*value	*P*-value^b^
		Positive	Negative						
ETRAMP4	Patients (50)	31	19	62.0/97.5	48.2–74.1	10,713 ± 10,787	52,033	4.341	< 0.0001
	Healthy (40)	1	39		87.1–99.6	3276 ± 963.4			
ETRAMP5	Patients (50)	24	26	48.0/95.0	34.8–61.5	6115 ± 5629	4579	3.877	0.0002
	Healthy (40)	2	38		83.5–98.6	2620 ± 979.4			
ETRAMP8	Patients (50)	23	27	46.0/95.0	32.8–59.6	6678 ± 6376	4589	3.600	0.0005
	Healthy (40)	2	38		83.5–98.6	3023 ± 783.1			
ETRAMP9	Patients (32)	14	18	43.8/100	28.2–60.7	4428 ± 2008	3968	2.015	0.05
	Healthy (8)	0	8		67.6–100	2974 ± 497.2			
ETRAMP10.3	Patients (50)	20	30	40.0/97.5	27.6–53.8	5880 ± 5540	4259	3.643	0.0005
	Healthy (40)	1	39		87.1–99.6	2659 ± 799.8			
ETRAMP11.2	Patients (56)	43	13	76.8/97.5	64.2–85.9	12,647 ± 3326	9733	10.39	< 0.0001
	Healthy (40)	1	39		87.1–99.5	6811 ± 1461			

^a^Cut-off, the mean fluorescence intensity of the malaria-naïve samples plus 2 standard deviations

^b^*P-*value, the difference in the total IgG level for each antigen between *P. vivax* malaria patients and healthy individuals was calculated with the unpaired *t*-test. A *P-*value of < 0.05 was considered statistically significant

*Abbreviations*: CI, confidence interval; MFI, mean fluorescence intensity

### Subcellular localization of PvETRAMP4 in blood-stage parasites

**Fig. 4:**
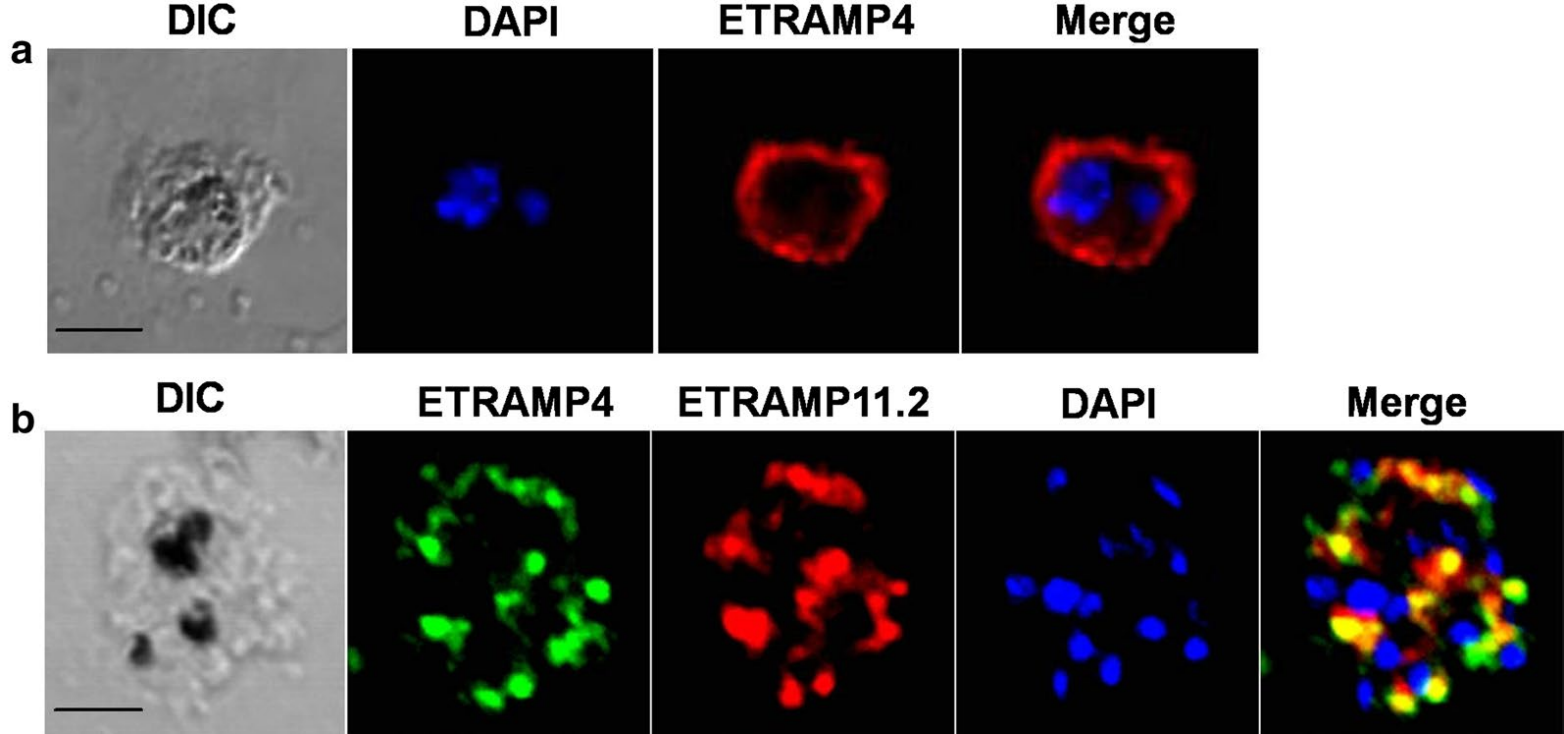
Localization and expression levels of PvETRAMP4 in *Plasmodium vivax* isolates. Parasites were stained with antisera against PvETRAMP4 (green color) PvETRAMP11.2 (red color) and DAPI (blue color). **a** Immature schizont stage. **b** Fully mature schizont stage. *Scale-bars*: 5 µm

Mice were immunized with a recombinant PvETRAMP4 protein for further analysis, and the specific serum reacted with the *P. vivax* isolate (Fig. 4). Interestingly, PvETRAMP4 showed signal associated membrane and organelle in different stages, and PvETRAMP11.2 partially colocalized with PvETRAMP4.

### Isotype prevalence of the antibody response in immunized mice

**Fig. 5:**
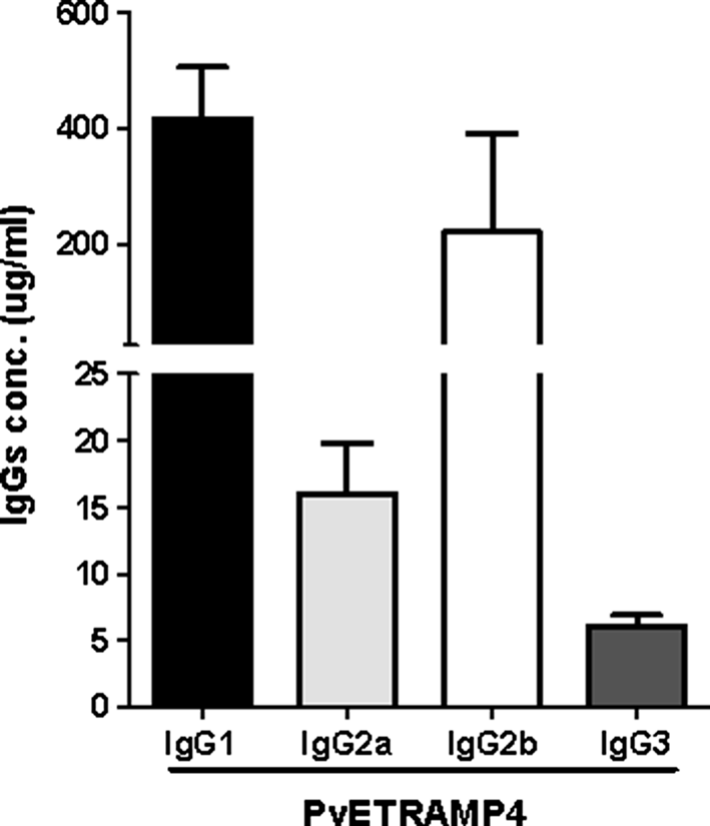
IgG subclass levels in response to PvETRAMP4 in immunized mice. The mice immunized with PvETRAMP4 were examined for the characterization of IgG subclasses. Error bars indicate the mean ± SD

To evaluate isotype prevalence, an anti-PvETRAMP4 antibody from immunized mice was analyzed by ELISA. As shown in Fig. 5, the major components of the antibody response against PvETRAMP4 were IgG1 and IgG2b. There was no significant difference between the levels of IgG2a and IgG3.

## Discussion

The ETRAMP family is distributed in all human-infectious malaria species as well as other mammalian malaria parasites, such as rodent and primate parasites. In addition, several ETRAMP family proteins are abundantly transcribed in the erythrocytic stages of *P. falciparum* and *P. vivax* [36, 37]; therefore, characterization and identification of the ETRAMP family are carried out for the development of serological markers and novel vaccine candidate discovery.

*Plasmodium vivax* has nine identifiable *etramps* genes, as shown in Table 2, and thus fewer than in *P. falciparum*, in which 13 ETRAMPs have been identified and found to be expressed at a specific stage [13]. Similar to *P. falciparum*, PvETRAMPs were predicted to be expressed specifically at the schizont and early ring stages. It is useful to identify the stage-specific proteins in the parasite life-cycle because they may play important and specific roles in parasite development. For example, the transcription levels of invasive proteins such as merozoite surface proteins were found to be sharply increased in the late schizont stage, indicating that the merozoites interact with erythrocytes through these proteins. In a previous report, PvETRAMP11.2 was considered to be an interaction partner of PvTRAg36.6, which is exported to the RBC membrane in transgenic *P. falciparum* and localizes to the apical ends of the free merozoites in *P. vivax*; additionally, the interaction between PvETRAMP11.2 and PvEXP1, which is considered to be essential for parasite growth, was confirmed [38, 39]. Moreover, the lower expression levels of four ETRAMPs found in *Saimiri* monkeys, to which the *P. vivax* parasite can infect, but without PvDBP-II binding, suggest that ETRAMPs expressed at a specific stage either directly or indirectly affect the regulation of functional protein(s) necessary for parasite growth [40].

One of the critical factors for the discovery and evaluation of potential targets that induce a strong humoral immune response in parasite-infected patients is the use of appropriate screening methods. In our previous study, a large number of *P. vivax* antigens were screened using a wheat germ cell free-expression system and protein array with *P. vivax* patient sera and healthy sera. A high correlation between the protein array and ELISA for the screening of serum reactivity was validated; therefore, we used the protein array method to analyze the humoral immune response against the ETRAMP family in *P. vivax* [41, 42]. Interestingly, PvETRAMP4 showed higher serum positivity than the other PvETRAMPs tested, although it does not seem to be directly exposed to the human immune system (Fig. 3, Table 3). Generally, the antigen that induces a humoral immune response in the host, such as EMP-1 or MSP-1, is a result of exposure to the immune system and is usually located on the erythrocyte or merozoite surface, which is continuously exposed to the human immune system. However, there is a possibility that some proteins within infected erythrocytes can also be exposed to the immune system upon lysis of the parasite after egress [43]. For a deeper understanding of the immune system response to PvETRAMP4, the IgG subclasses generated in immunized mice were evaluated. The IgG subclasses produced against PvETRAMP4 showed that IgG1 and IgG2b were predominantly produced. In a previous study, cytophilic isotypes (IgG1 and mainly IgG3) were found to be predominantly produced by *P. vivax* infection; IgG2b in mice plays a similar role to IgG3 in humans [44, 45], suggesting that PvETRAMP4 probably induces IgG3 in the human immune system. However, the immune response to other PvETRAMPs could not be evaluated due to a limited amount of protein. Therefore, there is a need to further study other ETRAMP members of *P. vivax*.

## Conclusions

For the immunological profiling of the *P. vivax* ETRAMP protein family, six proteins from the nine ETRAMP family proteins of *P. vivax* were expressed and purified, after which they were used for evaluation of the humoral immune response in *P. vivax* patients and immunization in mice. Interestingly, PvETRAMP4 and 11.2 showed relatively high antibody responses in *P. vivax* patients, and IgG isotype profiling showed that IgG1 and IgG2b were predominant in mice immunized with PvETRAMP4, suggesting that the PvETRAMPs might be candidates for vaccine and serological markers.

## Data Availability

The data supporting the conclusions of this article are included within the article.

## Abbreviations

ETRAMP: early transcribed membrane protein
SEP: small exported proteins
PCR: polymerase chain reaction
IgG: immunoglobulin G
PVM: parasitophorous vacuole membrane
RBC: red blood cell
PTEX: *Plasmodium* translocon for exported proteins (PTEX)
EXP-1: exported protein-1
MSP-1: merozoite surface protein 1
EMP-1: erythrocyte membrane protein-1

## References

[1] Reyes-Sandoval A, Bachmann MF (2013). *Plasmodium vivax* malaria vaccines: why are we where we are?. Hum Vaccin Immunother..

[2] WHO. World Malaria Report (2008–2018). Geneva: World Health Organization; 2018. https://www.who.int/malaria/publications/world_malaria_report/en/. Accessed 19 Nov 2018.

[3] Kumaratilake LM, Ferrante A (2000). Opsonization and phagocytosis of *Plasmodium falciparum* merozoites measured by flow cytometry. Clin Diagn Lab Immunol..

[4] Cohen S, Mc GI, Carrington S (1961). Gamma-globulin and acquired immunity to human malaria. Nature..

[5] Sabchareon A, Burnouf T, Ouattara D, Attanath P, Bouharoun-Tayoun H, Chantavanich P (1991). Parasitologic and clinical human response to immunoglobulin administration in falciparum malaria. Am J Trop Med Hyg..

[6] Cohen S, Butcher GA, Crandall RB (1969). Action of malarial antibody *in vitro*. Nature..

[7] McCallum FJ, Persson KE, Mugyenyi CK, Fowkes FJ, Simpson JA, Richards JS (2008). Acquisition of growth-inhibitory antibodies against blood-stage *Plasmodium falciparum*. PLoS One..

[8] Brown GV, Anders RF, Mitchell GF, Heywood PF (1982). Target antigens of purified human immunoglobulins which inhibit growth of *Plasmodium falciparum in vitro*. Nature..

[9] Miura K (2016). Progress and prospects for blood-stage malaria vaccines. Expert Rev Vaccines..

[10] Shen HM, Chen SB, Cui YB, Xu B, Kassegne K, Abe EM (2018). Whole-genome sequencing and analysis of *Plasmodium falciparum* isolates from China-Myanmar border area. Infect Dis Poverty..

[11] Bourgard C, Albrecht L, Kayano A, Sunnerhagen P, Costa FTM (2018). *Plasmodium vivax* biology: insights provided by genomics, transcriptomics and proteomics. Front Cell Infect Microbiol..

[12] Ntege EH, Takashima E, Morita M, Nagaoka H, Ishino T, Tsuboi T (2017). Blood-stage malaria vaccines: post-genome strategies for the identification of novel vaccine candidates. Expert Rev Vaccines..

[13] Spielmann T, Fergusen DJ, Beck HP (2003). etramps, a new *Plasmodium falciparum* gene family coding for developmentally regulated and highly charged membrane proteins located at the parasite-host cell interface. Mol Biol Cell..

[14] Vaughan AM, Aly AS, Kappe SH (2008). Malaria parasite pre-erythrocytic stage infection: gliding and hiding. Cell Host Microbe..

[15] Sharma A, Yogavel M, Akhouri RR, Gill J (2008). Crystal structure of soluble domain of malaria sporozoite protein UIS3 in complex with lipid. J Biol Chem..

[16] Kaiser K, Matuschewski K, Camargo N, Ross J, Kappe SH (2004). Differential transcriptome profiling identifies *Plasmodium* genes encoding pre-erythrocytic stage-specific proteins. Mol Microbiol..

[17] Mueller AK, Camargo N, Kaiser K, Andorfer C, Frevert U, Matuschewski K (2005). *Plasmodium* liver stage developmental arrest by depletion of a protein at the parasite-host interface. Proc Natl Acad Sci USA.

[18] Bano N, Romano JD, Jayabalasingham B, Coppens I (2007). Cellular interactions of *Plasmodium* liver stage with its host mammalian cell. Int J Parasitol..

[19] MacKellar DC, Vaughan AM, Aly AS, DeLeon S, Kappe SH (2011). A systematic analysis of the early transcribed membrane protein family throughout the life cycle of *Plasmodium yoelii*. Cell Microbiol..

[20] de Koning-Ward TF, Gilson PR, Boddey JA, Rug M, Smith BJ, Papenfuss AT (2009). A newly discovered protein export machine in malaria parasites. Nature..

[21] Johnson D, Gunther K, Ansorge I, Benting J, Kent A, Bannister L (1994). Characterization of membrane proteins exported from *Plasmodium falciparum* into the host erythrocyte. Parasitology..

[22] Simmons D, Woollett G, Bergin-Cartwright M, Kay D, Scaife J (1987). A malaria protein exported into a new compartment within the host erythrocyte. Embo J..

[23] Curra C, Di Luca M, Picci L, de Sousa Silva Gomes dos Santos C, Siden-Kiamos I, Pace T (2013). The ETRAMP family member SEP2 is expressed throughout *Plasmodium berghei* life cycle and is released during sporozoite gliding motility. PLoS One..

[24] Lu F, Li J, Wang B, Cheng Y, Kong DH, Cui L (2014). Profiling the humoral immune responses to *Plasmodium vivax* infection and identification of candidate immunogenic rhoptry-associated membrane antigen (RAMA). J Proteomics..

[25] Chen JH, Jung JW, Wang Y, Ha KS, Lu F, Lim CS (2010). Immunoproteomics profiling of blood stage *Plasmodium vivax* infection by high-throughput screening assays. J Proteome Res..

[26] Chuquiyauri R, Molina DM, Moss EL, Wang R, Gardner MJ, Brouwer KC (2015). Genome-scale protein microarray comparison of human antibody responses in *Plasmodium vivax* relapse and reinfection. Am J Trop Med Hyg..

[27] Jagannadham MV, Abou-Eladab EF, Kulkarni HM (2011). Identification of outer membrane proteins from an Antarctic bacterium *Pseudomonas syringae* Lz4W. Mol Cell Proteomics..

[28] Krogh A, Larsson B, von Heijne G, Sonnhammer EL (2001). Predicting transmembrane protein topology with a hidden Markov model: application to complete genomes. J Mol Biol..

[29] Sonnhammer EL, von Heijne G, Krogh A (1998). A hidden Markov model for predicting transmembrane helices in protein sequences. Proc Int Conf Intell Syst Mol Biol..

[30] Schultz J, Milpetz F, Bork P, Ponting CP (1998). SMART, a simple modular architecture research tool: identification of signaling domains. Proc Natl Acad Sci USA.

[31] Letunic I, Doerks T, Bork P (2009). SMART 6: recent updates and new developments. Nucleic Acids Res..

[32] Tsuboi T, Takeo S, Iriko H, Jin L, Tsuchimochi M, Matsuda S (2008). Wheat germ cell-free system-based production of malaria proteins for discovery of novel vaccine candidates. Infect Immunol..

[33] Chen JH, Wang Y, Ha KS, Lu F, Suh IB, Lim CS (2011). Measurement of naturally acquired humoral immune responses against the C-terminal region of the *Plasmodium vivax* MSP1 protein using protein arrays. Parasitol Res..

[34] Neeley ES, Kornblau SM, Coombes KR, Baggerly KA (2009). Variable slope normalization of reverse phase protein arrays. Bioinformatics..

[35] Cheng Y, Wang B, Sattabongkot J, Lim CS, Tsuboi T, Han ET (2014). Immunogenicity and antigenicity of *Plasmodium vivax* merozoite surface protein 10. Parasitol Res..

[36] Rutledge GG, Bohme U, Sanders M, Reid AJ, Cotton JA, Maiga-Ascofare O (2017). *Plasmodium malariae* and *P. ovale* genomes provide insights into malaria parasite evolution. Nature..

[37] Cui L, Fan Q, Hu Y, Karamycheva SA, Quackenbush J, Khuntirat B (2005). Gene discovery in *Plasmodium vivax* through sequencing of ESTs from mixed blood stages. Mol Biochem Parasitol..

[38] Tyagi K, Hossain ME, Thakur V, Aggarwal P, Malhotra P, Mohmmed A (2016). *Plasmodium vivax* tryptophan rich antigen PvTRAg36.6 interacts with PvETRAMP and PvTRAg56.6 interacts with PvMSP7 during erythrocytic stages of the parasite. PLoS One..

[39] Cheng Y, Lu F, Lee SK, Kong DH, Ha KS, Wang B (2015). Characterization of *Plasmodium vivax* early transcribed membrane protein 11.2 and exported protein 1. PLoS One..

[40] Gunalan K, Sa JM, Moraes Barros RR, Anzick SL, Caleon RL, Mershon JP (2019). Transcriptome profiling of *Plasmodium vivax* in *Saimiri* monkeys identifies potential ligands for invasion. Proc Natl Acad Sci USA.

[41] Chen JH, Chen SB, Wang Y, Ju C, Zhang T, Xu B (2015). An immunomics approach for the analysis of natural antibody responses to *Plasmodium vivax* infection. Mol Biosyst..

[42] Fan YT, Wang Y, Ju C, Zhang T, Xu B, Hu W (2013). Systematic analysis of natural antibody responses to *P. falciparum* merozoite antigens by protein arrays. J Proteomics..

[43] Horii T, Bzik DJ, Inselburg J (1988). Characterization of antigen-expressing *Plasmodium falciparum* cDNA clones that are reactive with parasite inhibitory antibodies. Mol Biochem Parasitol..

[44] Hussain R, Dawood G, Abrar N, Toossi Z, Minai A, Dojki M (1995). Selective increases in antibody isotypes and immunoglobulin G subclass responses to secreted antigens in tuberculosis patients and healthy household contacts of the patients. Clin Diagn Lab Immunol..

[45] Cheng Y, Shin EH, Lu F, Wang B, Choe J, Tsuboi T (2014). Antigenicity studies in humans and immunogenicity studies in mice: an MSP1P subdomain as a candidate for malaria vaccine development. Microbes Infect..

